# Closeness, Conflict, and Culturally Inclusive Pedagogy: Finnish Pre- and In-service Early Education Teachers’ Perceptions

**DOI:** 10.3389/fpsyg.2022.834631

**Published:** 2022-03-25

**Authors:** Wenwen Yang, Eero Laakkonen, Maarit Silvén

**Affiliations:** Department of Teacher Education, University of Turku, Turku, Finland

**Keywords:** culturally inclusive pedagogy, teacher self-efficacy, student–teacher relationship scale, measurement invariance, structural equation modeling, early childhood education

## Abstract

This study explored the factorial and concurrent validity of a scale developed for assessing teachers’ self-efficacy beliefs in engaging with diversity in early childhood education settings. According to tests of measurement invariance, the conceptualization of the constructs varied to some extent between Finnish student teachers and qualified teachers. Qualified teachers reported, at the item level, higher confidence in engaging with diversity in mainstream early childhood classrooms than student teachers. Structural equation modeling demonstrated that for both groups, higher levels of reported confidence in planning and implementing inclusive teaching–learning interactions were related to a higher level of closeness during interactions with children. The evidence for concurrent validity may imply beneficial and reciprocal influences between teachers’ confidence in their professional competence and close teacher–child relationships. The implications of the study are discussed from the perspective of teacher training and professional development in the early childhood education context.

## Introduction

According to the socio-cultural paradigm (e.g., Vygotsky, 1980; Bronfenbrenner, 1986; Rogoff, 2008), children’s development and learning are shaped by interactional experiences that are shared across generations in various cultural contexts. The growing global migration phenomenon has resulted in increasing ethnic, linguistic, and socio-economic diversity in Western societies, including European countries. Thus, children from various backgrounds are in critical need of teachers in daycare centers and schools who have been trained from an undergraduate level onward to be culturally competent and responsive and who can successfully and effectively interact with gender, age, ability, language, racial, and religious diversity in classrooms (Bandura, 1977; Pianta, 1999; Lieber et al., 2008; Ladson-Billings, 2009; Romaine, 2009; Gay, 2010; Sharma et al., 2012; Park et al., 2016; Gunn et al., 2021). Substantial evidence has shown that establishing high-quality teacher–child relationships in early childhood education (ECE) settings plays a fundamental role in children’s future development (e.g., Birch and Ladd, 1997; Pianta, 1999), but little is known about how teachers’ sense of efficacy in engaging with diversity in mainstream early childhood classrooms relates to teacher–child relationships in the ECE context. A deeper understanding of professional development is imperative for fostering optimal ECE for all children and improving ECE teacher education. The current study has two aims. First, the study attempts to bridge the research gap by developing a reliable and valid measure to assess teachers’ confidence in inclusive classroom practices. Given the likely differences in teachers’ professional competence and self-efficacy along their career-long development, we compare qualified ECE teachers’ and student teachers’ confidence in planning and implementing inclusive practices that recognize and respond to individual differences of all children. Second, the study explores the association between sense of efficacy for inclusive practices and the quality of teacher–child relationships, as perceived by the teachers.

### Self-Efficacy Beliefs

Over the past four decades, the role of self-efficacy beliefs in the context of teaching and learning has been explored to understand and support both novice and experienced teachers’ professional outcomes and behavioral changes. In Bandura’s (1977, 1997) social cognitive theory, self-efficacy is defined as teachers’ beliefs in their competence to organize and execute teaching to affect students’ learning outcomes regarding given attainments. Teachers’ expectations about their teaching competence, that is, efficacy beliefs, influence cognitive and emotional processes and act as a powerful motivational factor that can improve or undermine teachers’ actual performance with students (for reviews, see Tschannen-Moran and Woolfolk Hoy, 2001, 2007). Meta-analytic evidence provides some support for a reciprocal relationship between self-efficacy and performance (Honicke and Broadbent, 2016). A higher sense of efficacy may lead to greater efforts in actual teaching interactions and result in favorable learning outcomes for both the teacher and the students, which, in turn, strengthens teachers’ existing expectations about future success in teaching–learning interactions. A longitudinal study revealed that undergraduate students’ sense of efficacy improved from their first to the third and final year of study (Cassidy, 2012). Moreover, graduate students’ self-efficacy increased during the teacher preparation program characterized by gradual immersion into teaching but decreased after entering the profession as novice teachers (Woolfolk Hoy and Spero, 2005). Further, experienced teachers have a higher sense of self-efficacy compared to novice teachers (Tschannen-Moran and Woolfolk Hoy, 2007; for a cross-cultural study, see Malinen et al., 2013).

According to Bandura’s (1997) proposal, self-efficacy is context, task, and domain specific rather than a generalized construct. Several researchers have developed and validated scales that capture the multidimensional construct of teacher efficacy in general (see Gibson and Dembo, 1984; Bandura, 1997; Tshannen-Moran and Woolfolk Hoy, 2001). As confirmed by numerous studies conducted in the school context, teachers’ sense of efficacy varies across educational settings and subjects (for a review, see Tshannen-Moran and Woolfolk Hoy, 2001). There is a growing interest in studying teacher efficacy in inclusive practices as higher sense of efficacy has been identified as a crucial component in successful pedagogical interactions that are effective for students with diverse needs. In the current study, we employ a broad definition of diversity to include major developmentally significant dimensions, such as children’s ability, ethnicity, and language, which is in line with the inclusive nature of the Finnish educational system (see also Ainscow, 2016).

Cross-cultural studies on efficacy beliefs in teaching special needs students in the school context have applied the Teacher Efficacy for Inclusive Practices (TEIP) scale developed by Sharma et al. (2012), who provided evidence that the three-factor structure of TEIP scale fits pre-service teachers’ perceptions of efficacy in implementing inclusive practices across four countries (Canada, Australia, Hong Kong, and India). The three efficacy factors of the TEIP—use of inclusive instruction, collaboration, and managing behavior (after minor modifications)—were further validated in the school context with in-service teachers from China, Finland, and South Africa (Malinen et al., 2013). The factorial validity of the scale has also been explored by Park et al. (2016) in a sample of pre-service ECE teachers from the United States. The findings suggest that the factor structure of the TEIP scale is essentially unidimentional, with one higher-level general factor and the three more specific factors representing the three unique aspects of self-efficacy in implementing inclusive practices.

As maintained by the research literature in the United States, teachers’ cultural competence is connected to high-quality teaching interactions with ethnically diverse students in the school context (Ladson-Billings, 2009). According to Moule (2011), cultural competence is a set of congruent awareness, knowledge, and skills that enable professionals to be open minded to different world views and to connect with students from diverse cultures. Gay (2002, p. 106) defined culturally responsive teaching as “using the cultural characteristics, experiences, and perspectives of ethnically diverse students as conduits for teaching them more effectively”. Prior research has indicated that teachers who demonstrated culturally and linguistically responsive teaching practices were able to support positive school achievement and the social and language competence of children from diverse backgrounds (Ladson-Billings, 2009; Romaine, 2009; Gay, 2010; Ok et al., 2016; for ECE, see Han and Thomas, 2010; Gunn et al., 2021).

In a recent study on primary teachers’ self-efficacy on cultural competence conducted in the European Union (EU), Geerlings et al. (2018) showed that native Dutch in-service teachers experienced less self-efficacy with ethnic minority students compared to majority students. Moreover, self-efficacy in teaching children in heterogeneous classrooms proved to be positively associated with diverse classroom practices in a cross-national study by Romijn et al. (2020), based on a combined sample of ECE and primary teachers from four EU countries (Great Britain, Italy, the Netherlands, and Poland). The researchers assessed self-efficacy using a 7-item scale that included both general and diversity-specific items (see also Siwatu, 2007).

The foundation of language acquisition occurs from infancy to late preschool age (Bates et al., 1988; Silvén et al., 2014), which means that early childhood classrooms can be instrumental in supporting multilingual development. As language is deeply intertwined with culture and cultural identity, a responsive teacher must understand the special needs of children exposed to more than one language and possess the pedagogical knowledge and skills to support their multilingual growth and emerging confidence in their multicultural identity (Romaine, 2009; Lucas and Villegas, 2013). It can be argued that, similar to the school context, culture and language play an important role in teaching and learning in early childhood settings, and to maximize developmental outcomes, teachers need to embrace children’s diversity, motivate learning, and foster development through children’s lived experiences (see also Lieber et al., 2008; Han and Thomas, 2010; Gunn et al., 2021).

Surprisingly, few self-report scales have been specifically developed to assess ECE teachers’ cultural competence (for measures validated in the school context, see Spanierman et al., 2011; Yang et al., 2020; for Finnish, see Acquah et al., 2016). Among Hong Kong preschool teachers (> 98% Chinese) with varying educational backgrounds, Leung and Hue (2017) found a three-factor multicultural solution reflecting teaching skills, teaching knowledge, and teaching relationships. In a multi-ethnic sample of caregivers with varying educational backgrounds from the United States, Obegi and Ritblatt (2005) reported a one-factor solution for their cultural competence scale comprising awareness, knowledge, and skills items. Infant and toddler caregivers who had more education and training on the role of culture scored higher on cultural competence. At present, however, it is not clear whether the conceptualization of (multi)cultural competence in self-reports developed for culture-specific educational systems are generalizable across other educational, cultural, and linguistic contexts.

In the context of ECE, few studies have addressed the issue of teacher self-efficacy for inclusive practices that recognize and respond to the needs of all children with varied cognitive, emotional, and social abilities and familial backgrounds (for examples of inclusive practices in different EU countries).^1^ In the current Finnish study, we aimed to develop and validate a teacher efficacy scale that could provide new insights into how pedagogically confident ECE student teachers and qualified teachers are in engaging with diversity in early childhood classroom settings.

### Teacher–Child Relationships

An abundance of empirical research based on attachment theory and bio-ecological systems theory (Bowlby, 1969; Bronfenbrenner, 1986; Sroufe, 1988; Pianta, 1999) indicates that, similar to the development of trusting parent–child relationships in the early years, establishing a warm and positive teacher–child relationship has beneficial influences on children’s development (for reviews, see Sabol and Pianta, 2012; Groh et al., 2017; Neuhaus et al., 2020; Verschueren and Koomen, 2020). The findings are based on scales, interviews, and observation methods and show that higher quality teacher–child interactions are associated with better cognitive, social, and behavioral outcomes and less risk of school failure in the long term (Hamre and Pianta, 2001; Meehan et al., 2003; Stipek and Byler, 2004; Mashburn et al., 2006; Lippard et al., 2018). Among the different types of self-reports that are available for assessing the quality of teachers’ perceptions of their relational processes during classroom interactions in ECE settings, Pianta’s (2001a,b) 28-item Student–Teacher Relationship Scale (STRS) and its 15-item short form (STRS-SF) have gained worldwide acceptance. The original long form comprises subscales of closeness, conflict, and dependency. Closeness refers to a positive and warm relationship with a specific child, and conflict indicates negative and unpleasant feelings toward the child. The shortened form does not include dependency, which stands for a child’s overly dependent behavior.

A number of studies have examined the factorial validity of the STRS across different cultures, languages, and educational levels (for a review, see Tsigilis et al., 2018). The three- and the two-factor structure of the two forms have been validated in studies from United States and Europe. It seems that teachers’ responses to the STRS items depend on a combination of universal and culture-specific influences. The European findings suggest better-fitting three-factor models when certain STRS items are removed. Only the constructs of closeness and conflict, the two-factor structure of the STRS-SF, has been supported in the Northern European educational setting (Drugli and Hjemdal, 2013). Some studies have explored the measurement invariance of the STRS across child and contextual characteristics. The teachers in European studies seemed to interpret the meaning of STRS items similarly when assessing boys and girls, children of varying ages and in different educational contexts (Koomen et al., 2012; Milatz et al., 2014; Tsigilis et al., 2018). However, the three constructs of the STRS did not function uniformly for ECE teachers of ethnically different groups of children in the United States (Webb and Neuharth-Pritchett, 2011). So far, no studies have explored how ECE teacher characteristics, such as age, educational background, and professional experience, influence the measurement invariance of STRS.

The current study conducted in Northern Europe utilized a modified version of the STRS-SF (Whitaker et al., 2015), which estimates teachers’ perceptions of close and conflictual relationships with all children in the early childhood classroom rather than some individual children. A validation study on the two-factor structure of the modified version revealed that Finnish ECE student teachers and qualified teachers perceived themselves as having high levels of overall closeness and low levels of conflict during interactions in the classrooms, which characterize good relationships between the teacher and the children (Yang et al., 2021). The modified version of STRS-SF may be useful in teacher training to provide feedback about teachers’ pedagogical competence, to support on-going professional development, and to estimate the effect of training.

Regarding concurrent or predictive validity, researchers have used different types of aggregated versions of the STRS to examine the associations between teachers’ perceptions of relational processes and other major teacher characteristics, such as self-efficacy and depression, workplace stress, or educational contexts (Kesner, 2000; Hamre et al., 2008; Whitaker et al., 2015). Within the ECE setting in the United States, teachers’ sense of efficacy regarding the management and motivation of children (see Bandura, 1997) has been related to teachers’ perceptions of relational processes in the classroom (Mashburn et al., 2006; see also Hamre et al., 2008), children’s social competence (Mashburn et al., 2006), and language and literacy gains (Guo et al., 2010). In Finnish kindergarten classrooms, higher levels of teacher-perceived self-efficacy (see Gibson and Dembo, 1984) have been associated with higher levels of observed emotional support (Pakarinen et al., 2010). To sum up, teachers’ self-efficacy beliefs about their teaching competence and expectations for children may influence—and may be influenced by—the quality of their interactions in the ECE classrooms. Studies on primary school children suggest that classroom engagement mediates the association between affective teacher–child interaction and children’s later development (Kiuru et al., 2014; Roorda et al., 2017). Positive teacher–child relationships increase children’s engagement in learning rather than feelings of insecurity and allow the teachers to focus on optimal pedagogical practices for every child. Higher levels of stress and negative affect in the classroom may require the teacher to spend time with regulating the children’s emotional and motivational states at the expense of engaging in the teaching and learning process. Therefore, it is important to examine teachers’ efficacy in dealing with diversity in classrooms.

### Inclusive Education in the Finnish Context

The current study was conducted in a Northern European early childhood education and care (ECEC) context that has a long tradition of inclusive education in mainstream classrooms supported by the Ministry of Education and Culture. The values of the Finnish society—equity and equality—together with respect for diversity (for a discussion, see Ainscow, 2016) are reflected in ECEC legislation, which ensures affordable high-quality childcare services for all families with children ages 0–6 years (most infants are cared for at home). Moreover, every child under school age has a subjective right to attend ECEC, which comprises education, instruction, and care with a pedagogical emphasis on supporting the child’s wellbeing, balanced development, and learning, regardless of age, gender, language, ability, or other background. In line with the strong inclusive approach to educational practice, children with special needs and immigrant backgrounds attend mainstream classrooms.

In daycare centers, multi-professional teams, typically composed of three members with various levels of educational qualifications, are responsible for a group of 12 children (24 children if older than 3  years). The children–ECE staff ratio can be even smaller in mainstream classrooms with more diversity. The ECE teacher, who is the pedagogical leader of the team, must hold a bachelor’s degree in early childhood teacher education from a university. Before the latest legislation came into force, a bachelor’s degree in social sciences from an applied university also qualified for the position of an ECE teacher. The qualified ECE teachers in our study had a bachelor’s degree either from a university or an applied university and varying levels of experience from working in daycare centers, whereas the ECE student teachers were first-year undergraduates from a university.

The national core curriculum for ECEC (2018), determined by the Finnish National Agency for Education, steers the systematic evaluation, development, and implementation of high-quality and egalitarian ECEC throughout the entire country. It provides a common basis and instructions for developing the local curriculum for ECEC in municipalities and the individual ECEC plan for each child. The core curriculum addresses the importance of recognizing differing parental practices, home languages, religions, and worldviews among families. Moreover, the multi-professional team members are obligated to assess each child’s social, emotional, and cognitive needs and to regularly create and revise a pedagogical plan in collaboration with the parents to support the child’s development, stable relationships, and interaction with others. Further, children with special cognitive, emotional, or social needs are also supported in mainstream classrooms by special ECE teachers and professionals from social and healthcare services.

### The Present Study

To our knowledge, no previous studies have investigated how teachers’ sense of efficacy in engaging with diversity relates to teacher–child relationships in a Northern European ECE context that takes into account the strong inclusive approach in regular classrooms of the educational system. To fill this gap, there is a need to develop a psychometrically sound measure with an appropriate level of domain and cultural specificity for assessing the multifaceted construct of teachers’ inclusive pedagogy (see Tshannen-Moran and Woolfolk Hoy, 2001; Geerlings et al., 2018).

Our central research aim is to explore how confident ECE student teachers and qualified ECE teachers are in planning and executing inclusive practices that are effective for children with diverse needs in mainstream early childhood classrooms. According to Lucas and Villegas (2013) and Ross and Bruce (2007), learning how to teach children and students with diverse backgrounds should start in pre-service programs and continue throughout a teacher’s career. It is important to explore whether differences in inclusive pedagogy beliefs exist and what these differences entail to improve training from initial teacher preparation and beyond, and how such beliefs are related to teachers’ overall perceptions of relationships with children. Such knowledge may have implications for assessing professional development and improving the effectiveness of pre-service and in-service teacher training.

To fulfill the central goal of the study, we developed a Culturally Inclusive Pedagogy (CIP) scale and examined its psychometric characteristics, reliability, and factorial and concurrent validity. It has become increasingly clear that studies involving comparisons of group means or relationships within groups should explore whether the construct of interest shares the same meaning for different groups (see Meredith, 1993; Byrne, 2006; Little, 2013). Therefore, before drawing conclusions about mean differences and relationships, it is imperative to test for measurement invariance among undergraduates, novice teachers, and/or experienced teachers to ensure that the participants interpret the content of the items and the scale scores in the same way.

This study addresses the following research questions regarding the use of the CIP scale to assess the inclusive pedagogy beliefs of ECE student teachers and qualified ECE teachers: (1) What are the psychometric characteristics of the new CIP scale, including factor structure and measurement invariance? The construction of the CIP scale was built on Bandura’s (1977) description of self-efficacy and the Finnish national core curriculum for ECEC (Finnish National Agency for Education, 2018). The exploration of the factor structure was based on the assumption that successful engagement in planning and implementing inclusive practices can form a coherent one-factor structure (Hypothesis 1.1) or a two-factor structure, in which planning and implementing represent two independent but related factors (Hypothesis 1.2). (2) Are there differences in inclusive pedagogy beliefs across the two groups? Based on prior findings (Woolfolk Hoy and Spero, 2005; Tschannen-Moran and Woolfolk Hoy, 2007; Cassidy, 2012), we expect that experienced ECE teachers would have a higher sense of self-efficacy compared to undergraduate students during their first year of education (Hypothesis 2). (3) Do self-perceived teacher–child relational processes provide evidence for concurrent validity for the inclusive pedagogy beliefs for the two groups? Some evidence (Mashburn et al., 2006; Hamre et al., 2008) suggests that teachers’ sense of efficacy predicts the quality of interactions in the ECE classrooms (Hypothesis 3.1). In turn, the relationship may also be reciprocal (cp. Honicke and Broadbent, 2016) in that teachers’ self-efficacy may be influenced by the quality of interactions (Hypothesis 3.2).

## Materials and Methods

### Participants and Procedure

In the present study, we collected self-reports of ECE student teachers and ECE qualified teachers. The online questionnaire (Webropol) contained items about demographic characteristics, relationships with children, and culturally inclusive pedagogy. The participants took about 15–20 min to complete the questionnaire.

The ECE student teachers were first-year undergraduates (*n* = 171, females = 156) enrolled in a university bachelor’s degree program in early childhood teacher education (180 ECTS credits in 3 years; for more details about the European Credit Transfer and Accumulation System, see the European Commission’s website, https://ec.europa.eu). The curriculum of the degree program is composed of several study modules with different learning goals, content, and materials. Almost all students complete the curriculum modules in the recommended order. The questionnaire data were collected during two consecutive academic years at the beginning of a study module on ECE teacher’s pedagogical competence, which also included culturally and linguistically responsive teaching and inclusive pedagogy as one topic. Prior to filling out the questionnaire, the students had completed approximately 30 ECTS credits, including 1 week of practical training in daycare centers with 3–5-year-old children. In the 2019 cohort, of the 112 student teachers who responded to the questionnaire, 103 (93 females) gave their consent to use the self-reported data in the present study. In the 2020 cohort, 68 (63 females) of the 72 student teachers gave their consent.

The qualified ECE teachers (*n* = 155; females = 151) were working in daycare centers in two southwestern provinces of Finland during the study. The inclusion criterion for the ECE teachers was at least a bachelor’s degree in either education from a university or in social sciences from an applied university. We applied two different recruiting procedures. First, we sent invitations, which included an online Webropol questionnaire link, *via* email to the ECE administrators of 44 municipalities. The administrators were responsible for forwarding the invitations to the heads of daycare centers, who forwarded the emails to the qualified ECE teachers working in the center. Second, we also invited 40 qualified ECE teachers from eight different municipalities to a long-lasting in-service training based on up-to-date research-based knowledge and pedagogical skills arranged by a university. In Finland, teachers’ professional growth is seen as a continuum starting from pre-service training, and qualified teachers are expected to develop their professional competence throughout their careers. On the first in-service training day, 39 out of the 40 qualified ECE teachers filled out the online questionnaire (for more details, see Yang et al., 2021).

On average, the qualified teachers invited to in-service training had less working experience, *t*(113.47) = −3.05, *p* = 0.003, and worked with younger children, *t*(141) = −2.51, *p* = 0.013, in their classrooms compared to the qualified teachers who were not invited to participate in the in-service training. Otherwise, the two groups of qualified teachers did not differ in other aspects, such as educational background, child–teacher ratio, or the number of children in need of special support or from immigrant backgrounds in their classrooms. Regarding the educational backgrounds of the qualified teachers, 75 (48.4%) had a bachelor’s degree in social sciences from applied universities, 66 (42.6%) had a bachelor’s degree in ECE, and 14 (9%) had a master’s degree in ECE from universities. The age of the student teachers ranged from 19 to 49 years (*M* = 25.57, SD = 6.54), and the age of the qualified teachers ranged from 23 to 63 years (*M* = 42.84, SD = 11.14). Qualified teachers’ years of work experience ranged from 1 to 40 years (*M* = 14.25, SD = 10.64). The teachers worked with children between the ages of 1 and 7 years (*M* = 4.55, SD = 1.46). The number of children in need of special support and with immigrant backgrounds in the classrooms varied from 0 to 9 (*M* = 2.34, SD = 2.23) and from 0 to 22 (*M* = 3.50, SD = 4.52), respectively. The child–teacher ratio ranged from 2.33 to 12.00 (*M* = 5.73, SD = 1.77).

### Assessment of Teachers’ Culturally Inclusive Pedagogy Beliefs

The development of the CIP scale was based on Bandura’s (1977) construct of domain-specific efficacy expectations and existing operationalization discussed in the literature review. The ECEC legislation and the national core curriculum for ECEC (Finnish National Agency for Education, 2018) were the starting point for constructing the CIP items specific to the Finnish ECEC context. According to the curriculum (pages 8 and 35), ECE teachers have the overall responsibility for planning and implementing activities for groups of diverse children with a goal-oriented and systematic approach and in cooperation with parents. Two experts in ECE teacher education and practice in daycare centers developed a set of 16 items to assess pre- and in-service ECE teachers’ self-efficacy for planning and implementing inclusive educational practice in the mainstream ECE pedagogy. Half of the scale items were influenced by Siwatu’s (2007) self-efficacy scale constructed for teaching school children and validated among pre-service teachers. In contrast to existing diversity-specific scales (e.g., Siwatu, 2007; Park et al., 2016), we employ a broad definition of diversity and adjusted the content of the CIP items to include children’s ability, ethnicity, and language (see Table 1 for examples of the items and descriptive statistics).

**Table 1:** Culturally Inclusive Pedagogy scale items: descriptive statistics and reliability coefficients.

S. No.	Items	Student Teachers		Qualified Teachers	
		*M* (SD)	Range	*M* (SD)	Range
1.	I can take into account the diverse backgrounds of children when I am planning activities and interacting in teaching–learning interaction	3.95 (0.71)	2–5	4.00 (0.73)	2–5
2.	I can invent strategies to reduce conflict between the cultures of home and early childhood education	3.40 (0.80)	2–5	3.69 (0.74)	2–5
3.	I can help children from diverse backgrounds	3.83 (0.79)	2–5	3.89 (0.65)	2–5
4.	I can adjust my speech to children of diverse backgrounds during the teaching–learning interaction	3.85 (0.71)	2–5	4.19 (0.60)	3–5
5.	I can design the environment of early childhood education to take into account children from diverse backgrounds	3.75 (0.75)	2–5	3.86 (0.66)	2–5
6.	I can form groups of children in which children with diverse backgrounds interact positively with each other	3.77 (0.80)	2–5	4.07 (0.75)	2–5
7.	I can provide visual assistance for multilingual children	4.06 (0.80)	1–5	4.39 (0.70)	2–5
8.	In teaching–learning situations, I can invent examples that are suitable for children from diverse backgrounds	3.65 (0.93)	1–5	3.64 (0.84)	1–5

The reliability coefficient Rho based on the maximum likelihood estimation method of the 8-item CIP was 0.86 for the whole sample, 0.86 for the student teachers, and 0.84 for the qualified teachers. The Finnish version is available upon request from the authors.

For the qualified teachers, the questionnaire instructions stated as: “There are more and more children from different language, cultural, and family backgrounds in early childhood education. Consider the following statements and evaluate how well the statements describe your activities as an early childhood educator.” For student teachers with no stable classroom, we changed “as an early childhood educator” to “as a student teacher in early childhood education.” In the present study, participants rated each statement on a 5-point Likert scale (1 = *definitely does not apply* to 5 = *definitely applies*). A higher CIP score represents greater confidence in planning and implementing inclusive pedagogy.

### Assessment of Teachers’ Relationships With Children

We used a Finnish translation of the modified version (Whitaker et al., 2015) of the original STRS-SF (Pianta, 2001b) to estimate ECE student teachers’ and qualified teachers’ overall quality of their relationships with a group of children instead of one child. The content of the 15 items was the same in the modified version, except that the word “child” and the singular verbs had been changed to “children” and plural verbs. The modified version included the same two factors as the original STRS-SF (see Table 2 for examples of the items and descriptive statistics). Closeness represents the close, warm, and positive side and conflict represents the negative and conflictual side of teachers’ perceptions of relationships with children. The modified version has been shown to be a valid and reliable measure of relational processes, closeness, and conflict in the Finnish ECEC setting (Yang et al., 2021). The instructions were as: “Please assess how well each of the statements below currently applies to your relationship with children in your classroom. All relationships are individual, but in responding, please think about your relationships with the children in general. Use the scale below to choose the appropriate response for each item.” For the student teachers with no stable classroom, we used “children attending ECE settings” instead of “children in your classroom.” Each item was rated on a 5-point Likert scale ranging from 1 (*definitely does not apply*) to 5 (*definitely applies*). A higher score on Closeness represents closer relationships, and a higher score on Conflict indicates more conflictual relationships in the ECE classroom.

**Table 2:** Closeness and Conflict items: descriptive statistics and reliability coefficients.

S. No.	Items	Student Teachers		Qualified Teachers	
		*M* (SD)	Range	*M* (SD)	Range
1.	I share an affectionate, warm relationship with the children	4.72 (0.42)	3–5	4.77 (0.43)	3–5
2.	The children and I always seem to be struggling with each other	1.79 (0.77)	1–5	2.13 (0.82)	1–4
3.	If upset, the children will seek comfort from me	4.30 (0.62)	2–5	4.79 (0.42)	4–5
4.	The children value their relationship with me	4.30 (0.61)	3–5	4.48 (0.55)	3–5
5.	When I praise the children, they beam with pride	4.53 (0.60)	3–5	4.72 (0.50)	3–5
6.	The children share information with me about themselves even if I do not ask	4.53 (0.59)	3–5	4.47 (0.66)	2–5
7.	The children easily become angry with me	1.82 (0.72)	1–5	2.07 (0.94)	1–5
8.	The children are angry or do not care if they have been told off	2.18 (0.76)	1–5	2.25 (1.00)	1–5
9.	When the children are in a bad mood, I know we are in for a long and difficult day	2.73 (0.94)	1–5	2.71 (1.05)	1–5
10.	The children’s feelings toward me can be hard to predict or can change suddenly	1.91 (0.77)	1–5	2.08 (1.11)	1–5
11.	The children are sneaky or manipulative with me	1.40 (0.59)	1–4	1.36 (0.60)	1–5
12.	The children openly share their feelings and experiences with me	4.43 (0.55)	3–5	4.41 (0.59)	3–5

The reliability coefficient Rho based on the maximum likelihood estimation method of the 12-item Closeness and Conflict scales were 0.60 and 0.66 for the whole sample, 0.65 and 0.61 for the student teachers, and 0.56 and 0.70 for the qualified teachers. The Finnish version is available upon request from the authors.

## Results

### Statistical Analysis

We examined the factor structure of the CIP scale using Mplus 8 (Muthén and Muthén, 2017) for the total sample (*N* = 326), and separately for the student teachers (*n* = 171) and the qualified teachers (*n* = 155). After testing measurement invariance of the CIP scale across the two groups, we compared whether the factor means and variances of the construct differed between the two groups. Then, we tested the factor structure and measurement invariance of the STRS-SF composed of Closeness and Conflict factors. Next, we explored the concurrent validity of the CIP scale by testing the three-factor measurement model (e.g., Schumacker and Lomax, 1996) based on Culturally Inclusive Pedagogy, Closeness, and Conflict factors. Lastly, we specified two structural models for exploring the reciprocal (predictive) relations between Culturally Inclusive Pedagogy and Closeness and Conflict separately for the two groups.

The parameters of the models were estimated using the maximum likelihood estimation method. The following indicators were used to evaluate the goodness-of-fit of the models to the covariance matrix of the sample data: the chi-square likelihood ratio test (*p* > 0.05), the comparative fit index (CFI ≥ 0.95), the Tucker–Lewis index (TLI ≥ 0.95), the standardized root mean squared residual (SRMR ≤0.08), and the root mean squared error of approximation (RMSEA ≤0.06; Hu and Bentler, 1999). To identify model misspecification, we inspected the theoretical meaningfulness and *t*-values of the estimated parameters, *R*^2^ values, standardized residuals, and modification indices (MIs). For the cross-group tests, the fit of an alternative nested model was determined with a chi-square difference test (Δ*χ*^2^) and improvement in other fit indices. A significant decrease in the chi-square difference and a comparative fit index difference greater than 0.01 suggested that the less restrictive model better fit the data than the more restrictive alternative model (Cheung and Rensvold, 2002).

The distribution of the sample data was within the normal range, with skewness below |2| and kurtosis below |7| (West et al., 1995). For the CIP scale and the STRS-SF scale, skewness ranged from −1.77 to 0.15 and from −1.32 to 1.76, and kurtosis from −0.73 to 2.31 and from −0.80 to 4.64, respectively. The missing data were treated as missing at random, but there were very few missing items in the CIP scale (0.04%) and in the STRS-SF scale (0.08%), suggesting that the risk for biased parameter estimates due to missing data was minimal.

Most of the correlations (0.18–0.57) for the 16-item CIP were positive and significant. Two CIP items (“I can praise the child in their native language.” and “I appreciate children’s diverse backgrounds.”) were excluded from the subsequent confirmatory factor analysis (CFA) due to non-significant and low correlations (<0.31) with most other items of self-efficacy for planning and implementing inclusive educational practice. As expected, for the 15-item STRS-SF, most of the correlations were positive and significant between the closeness items (0.15–0.42) and between the conflict items (0.14–0.43).

### Factor Structure of Culturally Inclusive Pedagogy

Multi-group CFA was conducted to examine the fit of the postulated one-factor model of the 14-item CIP scale (Hypothesis 1.1). The goodness-of-fit indices were unacceptable, and only after removing the six items with the lowest factor loadings one by one, we ended up with an 8-item solution with acceptable model fit for the whole sample as well as for the qualified teachers. For the student teachers, all fit indices were acceptable except for the significant chi-square value. We noticed that items 3 and 4 have overlapping meaning, and this overlap in content may cause a need for correlated error terms, which was also confirmed by the model MIs. Adding an error covariance between the two items to indicate the method effect gave all three models an acceptable fit (Table 3). All standardized factor loadings were significant (*p* < 0.001) and ranged from moderate to strong (whole sample = 0.57–0.74, student teachers = 0.59–0.74, and qualified teachers = 0.49–0.76). The 8-item CIP (see Table 1 for the items and the reliability coefficients) assesses how confident teachers perceived themselves when planning activities for children with diverse backgrounds (items 1, 5, and 6) and home cultures (item 2), and implementing activities and interacting with diverse children (items 3, 4, 7, and 8).

**Table 3:** Fit indices of Culturally Inclusive Pedagogy: one-factor model.

Sample	*χ* ^2^	df	*p*	CFI	TLI	RMSEA	SRMR
Whole sample (*N* = 326)	21.43	19	0.314	0.997	0.996	0.020	0.023
Student teachers (*n =* 171)	23.51	19	0.216	0.991	0.987	0.037	0.033
Qualified teachers (*n* = 155)	9.73	19	0.959	1.00	1.00	<0.001	0.022

Next, we tested the fit of the two-factor model of the 14-item CIP scale based on the assumption that self-efficacy for inclusive educational practice contains two separate but intertwined constructs: self-efficacy for planning and implementing (Hypothesis 1.2). In addition, the CIP contained three items related to home culture and collaboration with parents, which can relate to both constructs. We therefore explored two versions of the two-factor structure, forcing the items first in the planning factor and then in the implementing factor. Again, the goodness-of-fit indices were unacceptable for both two-factor models. After deleting in both models the same four items with the lowest factor loadings one by one and adding one error covariance (between items 8 and 9, not shown in Table 1), we found acceptable model fit of both two-factor models for the whole sample and for the qualified teachers (Table 4). Adding one more error covariance (between items 3 and 4) gave all three models an acceptable fit.

**Table 4:** Fit indices of Culturally Inclusive Pedagogy: two-factor model.

Sample	*χ* ^2^	df	*p*	CFI	TLI	RMSEA	SRMR
**Model (A) Planning and implementing (including home culture)**							
Whole sample (*N* = 326)	36.29	32	0.276	0.996	0.994	0.020	0.024
Student teachers (*n =* 171)	45.23	32	0.061	0.980	0.971	0.049	0.037
Qualified teachers (*n* = 155)	20.59	32	0.972	1.00	1.00	<0.001	0.028
**Model (B) Planning (including home culture) and implementing**							
Whole sample (*N* = 326)	35.91	32	0.290	0.996	0.995	0.019	0.024
Student teachers (*n =* 171)	44.32	32	0.072	0.981	0.973	0.047	0.036
Qualified teachers (*n* = 155)	20.47	32	0.943	1.00	1.00	<0.001	0.029

To select which of the competing models best explains the data for the whole sample, we used the Akaike information criterion (AIC), the Bayesian information criterion (BIC), and the sample size adjusted BIC (adjBIC). The one-factor model had the lowest values (AIC = 5104.82, BIC = 5199.49, and adjBIC = 5120.20) compared to the two-factor models (Model a: AIC = 6402.50, BIC = 6527.46, and adjBIC = 6422.79; Model b: AIC = 6402.12, BIC = 6527.09, and adjBIC = 6422.41). The correlations between the two factors (>0.95) were very high, suggesting that they shared a high proportion of their variance (i.e., poor discriminant validity). Furthermore, all the CIP items of the one-factor model were included in the two-factor models. Based on these reasons, we selected the one-factor structure over the two-factor structure.

### Measurement Invariance of Culturally Inclusive Pedagogy

Table 5 presents the results of the sequence of increasingly more restrictive models for testing measurement invariance with multi-group CFA across the groups of ECE student teachers and qualified teachers (for the testing procedure, see Meredith, 1993; Byrne, 2006). Configural invariance is the least restrictive level and tests whether the factor structure and patterns of factor loadings of a model are similar across groups. As shown by the fit statistics of the configural model (M_1_), the one-factor structure of Culturally Inclusive Pedagogy and patterns of factor loadings fit the data for both student teachers and qualified teachers.^2^ The next more restrictive level tests for metric invariance by constraining the magnitude of the factor loadings to be equal across the two groups. When comparing the metric invariance model (M_2_) with the configural baseline model (M_1_), in which the one-factor structure was held invariant, the non-significant result of the chi-square difference test and the other fit statistics supported the assumption of an invariant pattern of factor loadings. This indicates that the factor loadings were equally strong indicators of the Culturally Inclusive Pedagogy construct among both the student teachers and qualified teachers. Attaining configural as well as metric invariance demonstrated weak measurement invariance that was not sufficient for reliable group comparisons (Meredith and Teresi, 2006; Little, 2013).

**Table 5:** Fit indices of measurement invariance models of Culturally Inclusive Pedagogy.

Model (M)	*χ* ^2^	df	CFI	TLI	RMSEA	Δ*χ*^2^-test (df)	*p*	ΔCFI
M_1_: Configural	33.24	38	1.00	1.00	<0.001		0.689	
M_2_: Metric	37.42	45	1.00	1.00	<0.001	4.18 (7)	0.759	<0.001
M_3_: Scalar	83.01	52	0.964	0.961	0.060	45.59 (7)	<0.001	0.036
M_4_: Partial scalar (4 intercepts free)	39.48	48	1.00	1.00	<0.001	2.06 (3)	0.561	<0.001
M_5_: Partial scalar equal means	40.32	49	1.00	1.00	<0.001	0.85 (1)	0.358	<0.001
M_6_: Partial scalar equal variances	42.35	49	1.00	1.00	<0.001	2.88 (1)	0.090	<0.001
M_7_: Partial scalar (2 intercepts free)	58.45	50	0.990	0.989	0.032	21.03 (5)	0.001	0.010
M_8_: Partial scalar equal means	62.43	51	0.987	0.985	0.037	3.98 (1)	0.046	0.003
M_9_: Partial scalar equal variances	61.29	51	0.988	0.987	0.035	2.84 (1)	0.092	0.002

To test the next restricted level of scalar invariance, the item intercepts were constrained as equal across the two groups (M_3_). As depicted in Table 5, the results revealed a statistically significant decrease in model fit, thus indicating that the estimated parameters (the constrained intercepts) were unequal across groups, which suggests that strong measurement invariance was not achieved. Inspection of the MI and output suggested that, compared to the students teachers, the qualified teachers scored higher on items 2 (“I can invent strategies to reduce conflict between the cultures of home and early childhood education.”), 4 (“I can adjust my speech to children of diverse backgrounds during the teaching–learning interaction.”), 6 (“I can form groups of children in which children with diverse backgrounds interact positively with each other.”), and 7 (“I can provide visual assistance for multilingual children.”). Hence, the intercepts of the four non-invariant items were set free one by one to take on any value to produce a partial scalar invariance model (M_4_). The non-significant chi-square difference as well as reasonably acceptable change in CFI (<0.01) provided adequate support for the partial scalar invariance (M_4_) across student teachers and qualified teachers. Success in passing at least partial scalar invariance is a prerequisite for comparing factor means and factor variances between different groups (Meredith and Teresi, 2006; Little, 2013).

### Group Comparison of Culturally Inclusive Pedagogy

After testing the measurement invariance of the observed variables, we explored factor-level differences in sense of self-efficacy across groups (Hypothesis 2). We compared the equality of the means and variances of the Culturally Inclusive Pedagogy factor across student teachers and qualified teachers (see Table 5). First, the model with equality constraints on the factor means (M_5_) across groups did not differ from the partial scalar invariance model (M_4_), as suggested by the non-significant decrement in the chi-square difference test. In contrast to our expectation, the qualified teachers reported equally high self-efficacy beliefs regarding culturally inclusive pedagogy (factor mean = 4.01, 95% CI = 3.91–4.10) as the student teachers (factor mean = 3.95, 95% CI = 3.85–4.05). Second, as suggested by the non-significant decrement in the chi-square difference test, the model with equality constraints on the factor variance (M_6_) did not differ from the partial scalar invariance model (M_4_). The ECE student teachers’ and qualified teachers’ beliefs regarding culturally inclusive pedagogy showed equal variability (factor variances were 0.27 and 0.20, respectively).

As demonstrated by Chen (2008, see also Byrne et al. 1989), the proportion of non-invariant items in a scale can bias the factor means for the two groups. To explore the magnitude of the bias in factor means, we reran the models M_4_, M_5_, and M_6_ in Table 5 by setting free only two instead of four non-invariant item intercepts (M_7_, M_8_, and M_9_). As expected, the factor mean was inflated for the qualified teachers (factor means = 4.04, 95% CI = 3.94–4.13) and deflated for the student teachers (factor means = 3.92, 95% CI = 3.82–4.02). Moreover, the significant decrement in the chi-square difference test suggested that the qualified teachers reported higher self-efficacy beliefs regarding culturally inclusive pedagogy than the student teachers. To further explore group differences, we calculated a summary score across the eight CIP items and conducted an independent-samples *t*-test. The results, *t*(323) = −3.14, *p* = 0.002, showed that the qualified teachers (*M* = 3.97, SD = 0.49) reported higher self-efficacy beliefs compared to student teachers (*M* = 3.78, SD = 0.57).

### Factor Structure and Measurement Invariance of Closeness and Conflict

Before exploring concurrent validity, we used multi-group CFA to determine whether the hypothesized two-factor structure of the Finnish 12-item version of the STRS-SF fits the sample data (see Table 2, for the items and the reliability coefficients). Due to item content overlap, we allowed for three error covariances (between items 6 and 12, items 9 and 10, and items 10 and 11) as previously suggested (Yang et al., 2021). Adding error covariances is a typical way to improve the model fit due to a degree of overlap in item content (Byrne, 2006). The model fits were acceptable for the student teachers and the qualified teachers but not for the whole sample (see Table 6). All standardized factor loadings were significant (*p* < 0.05) and ranged from low to high (whole sample = 0.28–0.68, student teachers = 0.32–0.64, and qualified teachers = 0.19–0.79). Although some loadings are fairly low, most loadings were at least over the conventionally acceptable level (0.30). The standardized correlations between Closeness and Conflict for the whole sample, student teachers, and qualified teachers were − 0.51, −0.58, and − 0.53, respectively. Taken together, the measurement model for Closeness and Conflict replicated our prior findings reported for a smaller student sample (Yang et al., 2021).

**Table 6:** Fit indices of Closeness and Conflict: factor structure models.

Sample	*χ* ^2^	df	*p*	CFI	TLI	RMSEA	SRMR
Whole sample (*N* = 326)	76.92	50	0.009	0.955	0.941	0.041	0.045
Student teachers (*n =* 171)	66.42	50	0.060	0.941	0.922	0.044	0.057
Qualified teachers (*n* = 155)	57.98	50	0.205	0.975	0.967	0.032	0.051

The results of testing the measurement invariance of the STRS-SF across the two groups are shown in Table 7. The configural model (M_1_) fits the data fairly well, suggesting that the pattern of items representing the constructs of Closeness and Conflict is similar for the student teachers and the qualified teachers.^2^ However, the fit indices of the metric model (M_2_) revealed that the assumption of equivalent factor loadings across groups must be rejected. Failure to achieve metric invariance (i.e., weak measurement invariance) demonstrated that ECE student teachers and qualified teachers interpreted at least some of the indicators of Closeness and Conflict differently, which is somewhat different from our prior evidence (Yang et al., 2021).

**Table 7:** Fit indices of Closeness and Conflict: measurement invariance models.

Model (M)	*χ* ^2^	df	CFI	TLI	RMSEA	Δ*χ*^2^-test (df)	*p*	ΔCFI
M_1_: Configural	124.40	100	0.959	0.946	0.039		0.050	
M_2_: Metric	151.71	110	0.931	0.917	0.048	27.31 (10)	0.002	0.028

### Concurrent Validity of Culturally Inclusive Pedagogy

Next, we used multi-group CFA to explore the concurrent validity of the CIP scale and the reciprocal (predictive) relations between teachers’ self-efficacy and self-rated relational processes. The fit indices for the measurement models with three correlated factors (Closeness, Conflict, and Culturally Inclusive Pedagogy) indicated good fit (see Table 8), with the exception of the chi-square tests, which revealed that the model fit was unacceptable for the whole sample but was more appropriate for the qualified teachers than the student teachers, as suggested by the *p* value at the threshold for acceptable fit (0.049 < 0.050). Although all standardized factor loadings were significant, the loadings, as expected, were somewhat lower for Closeness and Conflict compared to Culturally Inclusive Pedagogy. The correlations between Culturally Inclusive Pedagogy and Closeness were 0.57 and 0.55, between Culturally Inclusive Pedagogy and Conflict were − 0.39 and − 0.14, and between Closeness and Conflict were − 0.60 and − 0.53 for the student teachers and qualified teachers, respectively. All the correlations were significant (*p* < 0.001) except for Cultural Inclusive Pedagogy and Conflict among the qualified teachers (*p* = 0.16).

**Table 8:** Fit indices of concurrent validity: three-factor measurement models.

Sample	*χ* ^2^	df	*p*	CFI	TLI	RMSEA	SRMR
Whole sample (*N* = 326)	228.96	163	0.001	0.959	0.952	0.035	0.046
Student teachers (*n =* 171)	193.90	163	0.049	0.963	0.957	0.033	0.058
Qualified teachers (*n* = 155)	182.45	163	0.142	0.973	0.969	0.028	0.058

The structural models specified the hypothetical bidirectional relationships between the three factors. Instead of using multi-group CFA, we explored the direct effects of Culturally Inclusive Pedagogy on Closeness and Conflict (Hypothesis 3.1) and the reversed direction (Hypothesis 3.2) separately for the ECE student teachers and qualified teachers. This choice was motivated by the failure to achieve even weak measurement invariance for the STRS-SF and also by the unacceptable model fit (chi-square test) across the whole sample. When the predictors lack factor loading invariance, the regression slope can be underestimated in one group but overestimated in the other group (Chen, 2008; see also Borsboom, 2006).

**Figure 1:**
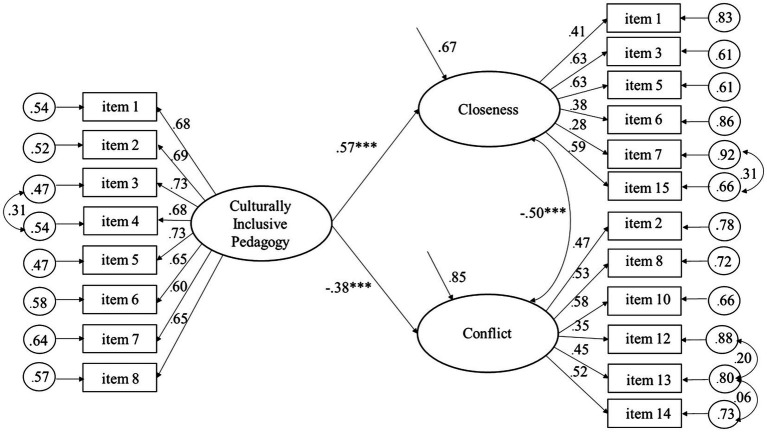
Predicting Closeness and Conflict from Culturally Inclusive Pedagogy (completely standardized solution) for the student teachers. ^***^p < 0.001.

**Figure 2:**
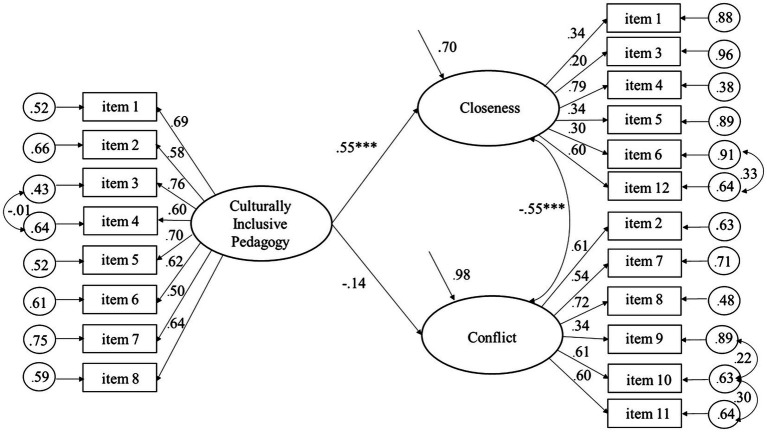
Predicting Closeness and Conflict from Culturally Inclusive Pedagogy (completely standardized solution) for the qualified teachers. ^***^p < 0.001.

**Figure 3:**
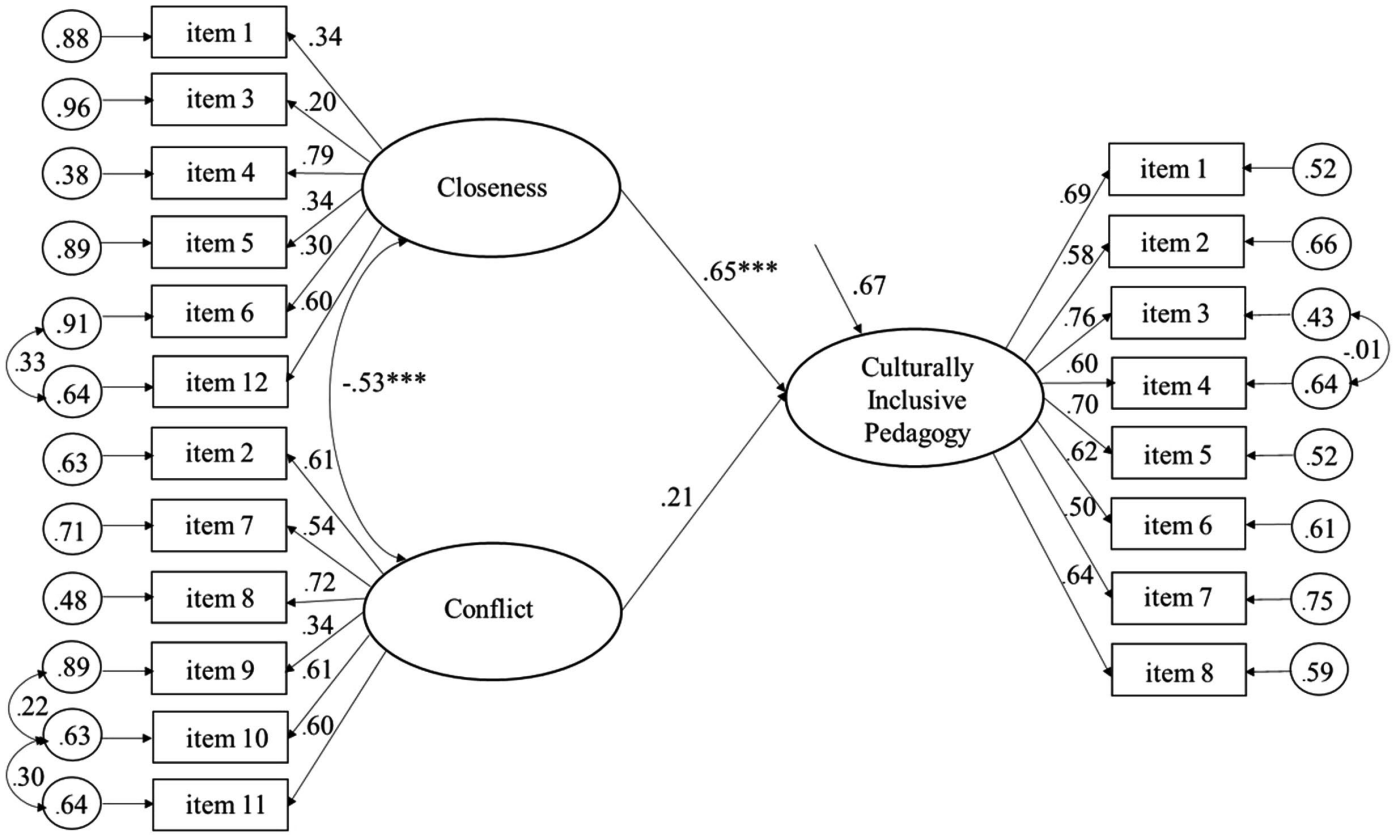
Predicting Culturally Inclusive Pedagogy from Closeness and Conflict (completely standardized solution) for the student teachers. ^***^p < 0.001.

**Figure 4:**
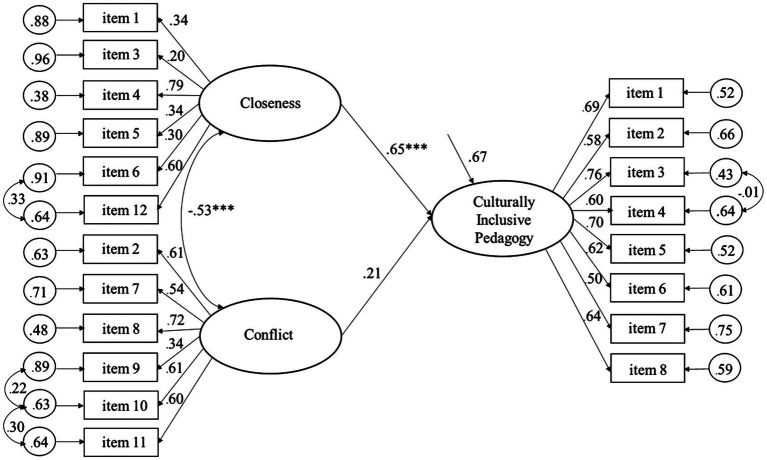
Predicting Culturally Inclusive Pedagogy from Closeness and Conflict (completely standardized solution) for the qualified teachers. ***p < 0.001.

As shown by the significant regression coefficients in Figure 1, Culturally Inclusive Pedagogy predicted positively to Closeness and negatively to Conflict, explaining 32.8 and 14.6% of the variance, respectively, among the student teachers. Figure 2 shows that Culturally Inclusive Pedagogy was significantly related to Closeness (but not Conflict), explaining 29.7% of the variance among the qualified teachers. The findings are consistent with the pattern of correlations of the measurement models. Moreover, as shown by the significant regression coefficients in Figures 3, 4, Closeness was positively associated with Culturally Inclusive Pedagogy, explaining 33.1 and 32.7% of the variance among the student teachers and qualified teachers, respectively. In other words, the teachers who perceived themselves as having a warmer and closer relationship with the children also perceived themselves as pedagogically more confident and competent when interacting with children and families from diverse backgrounds. Conflict did not predict variation in Culturally Inclusive Pedagogy among the student teachers (*p* = 0.66) or the qualified teachers (*p* = 0.10).

## Discussion

The overarching purpose of the current Finnish study was to explore the factorial and concurrent validity of the CIP scale developed for assessing teacher self-efficacy of culturally inclusive pedagogy. The current study contributes to a greater understanding of pre- and in-service ECE teachers’ sense of efficacy and the importance of establishing measurement invariance before making meaningful group comparisons. It seems justifiable to conclude that the qualified ECE teachers felt more confident and competent, at least on the item level, in engaging with diversity in mainstream classrooms than the first-year ECE student teachers. It should be noted that the underlying construct of self-efficacy was to some extent differently conceptualized by the two groups. The evidence for concurrent validity suggests that teachers’ confidence in their professional competence already during initial teacher education can have beneficial reciprocal influences on the quality of their relationships with children.

In line with the tenet that the self-efficacy of pedagogical competence is context-specific (Bandura, 1997; Tschannen-Moran and Woolfolk Hoy, 2007), the CIP scale was developed to reflect the policy of inclusive educational practice typical, for example, in North European countries. The inclusion of children with various abilities and familial backgrounds is common practice in mainstream Finnish classrooms. The items were developed by Finnish experts to contain two highly intertwined facets of ECE teachers’ self-efficacy beliefs. Multi-group CFAs confirmed that both a one-factor and a two-factor model fit the whole sample and the two groups (student teachers and qualified teachers) separately. The unidimensional construct of efficacy reflects in a more parsimonious way confidence in planning and implementing inclusive practice with diverse children.

A comparison of the factor means and variances interestingly revealed that ECE student teachers perceived themselves as being equally confident and pedagogically competent in engaging with diversity in early childhood classroom settings as qualified ECE teachers, despite the latter’s greater working experience. Similarly, the amount of variability in self-efficacy did not differ between the two groups. The factor-level findings could partly indicate shared values and beliefs in professional competence rather than first-year undergraduate students’ actual competence based on experience with diversity adopted during initial teacher education. This interpretation is consistent with evidence that students’ sense of self-efficacy tends to increase during teacher education when immersion into teaching is gradual but falls when they enter into their independent teaching profession (Woolfolk Hoy and Spero, 2005).

As mastery experience is the most potent source of self-efficacy (Bandura, 1997), it would be reasonable to expect a sense of self-efficacy to increase with the length of time spent in the profession, as demonstrated by Tschannen-Moran and Woolfolk Hoy (2007). Besides educational level and years of working experience, age can be considered to some extent as a proxy of experience in teaching. Additional analyses of our data suggested that age alone was a significant predictor of sense of self-efficacy both among student teachers (β = 0.25) and qualified teachers (β = 0.25). The qualified ECE teachers in our study were well educated, each having a minimum of a bachelor’s degree. The sample of ECE student teachers was mainly composed of more or less fresh graduates from high schools, but it also included older students, some of whom had other vocational qualifications or working experience in daycare centers. Given the qualified teachers’ educational levels and working experience (ranging from 1 to 40 years) compared to student teachers, one would expect more experienced teachers to have more professional confidence and a more profound understanding of their relationships with children and parents from diverse backgrounds.

Our study fills a research gap that has been overlooked in studies comparing pre- and in-service teachers (e.g., Spanierman et al., 2011; Ismailos et al., 2019) by rigorously testing different degrees of measurement invariance (Meredith, 1993; Little, 2013). According to the stringent guidelines for making valid and meaningful group comparisons, a researcher should establish strong, or at least partially strong, measurement invariance for a scale. Careful testing of measurement invariance revealed that the meanings of four items of the CIP scale were interpreted differently by the two groups of teachers. The qualified teachers, on average, scored higher on all four non-invariant items than the first-year student teachers. The item-level differences reflect variation in pedagogical practices, such as adjusting speech and providing visual assistance to diverse children, forming groups of children who interact positively with each other, and communicating with parents to reduce conflict between the home and ECE setting. Research by Chen (2008) demonstrated that as the proportion of non-invariant items increased, the degree of bias in factor means increased accordingly. Hence, to further understand the influence of intercept non-invariance, we reran the CFAs while allowing only 25% (instead of 50%) of the intercepts to vary. The analyses revealed that the differences in the observed variables were now reflected in the factor mean difference in favor of the qualified teachers, who reported higher self-efficacy for culturally inclusive pedagogy than the student teachers.

To provide evidence of concurrent validity of the CIP scale with well-established scales on self-perceived relational processes, we used the STRS-SF, which has shown cross-cultural robustness and acceptable psychometric properties, including in Northern European educational contexts. The measurement and structural models reflecting the reciprocal relationships between the constructs were run separately for the first-year ECE student teachers and qualified ECE teachers (for justification, see Borsboom, 2006; Chen, 2008). The models demonstrated that the more pedagogically efficacious the participants in both groups felt when planning and engaging in teaching–learning interactions with diverse children, the closer and warmer relationships they perceived themselves as having with the children. The opposite prediction was equally confirmed as: closer relationships predicted variation in teacher self-efficacy of culturally inclusive pedagogy. Our findings provide further support for the study by Mashburn et al. (2006), who found that ECE teachers’ efficacy regarding management and motivation of children were positively related with closeness but not relational conflict. Only among first-year ECE student teachers did higher self-efficacy predict greater closeness as well as less conflict in their relationships with children representing diverse abilities and various ethnic, linguistic, and other familial backgrounds. Our findings extend previously reported evidence on qualified ECE teachers (e.g., Mashburn et al., 2006; Hamre et al., 2008) and highlight the need to further explore the role of conflictual relationships in professional development. Thus, it is important to obtain validation of the reciprocal effects between teacher efficacy and relational processes by using longitudinal research designs.

The following limitations should be addressed in future research. First, reliable and valid self-reports are valuable screening tools, but due to the nature of self-reports, participants may respond in a way they believe to be socially desirable. Therefore, researchers should include multiple approaches, in-depth interviews, and observations of participants’ pedagogical interactions with diverse children to determine the extent to which self-perceptions and beliefs are consistent with practices in ECE classrooms. Second, the current study made use of robust and advanced statistical tools, but the sample size (*N* = 326) did not allow us to conduct an EFA within each subsample or subsequent CFA cross-validation analysis. Our analyses consisted of rather basic and typical CFA- and CFA-based modellings, which are not especially complex, so we evaluated the sample size as large enough. Third, all univariate distributions were within the reasonable limits suggested by West et al. (1995), but tests for univariate and multivariate normality detected some non-normality. Nevertheless, the analyses were conducted under the assumption of multivariate normality because the normality tests are well known to be sensitive to even slight departures from normality. Furthermore, the maximum likelihood estimation, which was used in the analyses, is known to have a degree of robustness to multivariate non-normality (e.g., Brown, 2015). Fourth, the psychometric properties of the Closeness and Conflict factors (some relatively low factor loadings and reliability coefficients, and three error covariances) may indicate that the widely used STRS-SF scale captures the meaning of relational processes somewhat differently across cultures. From a cross-cultural perspective, researchers should be concerned with the balance between maintaining the common meaning of the core constructs of Closeness and Conflict while accepting slight modifications by either including or deleting some highly culture-specific items (see also Chen, 2008; Sabol and Pianta, 2012).

According to Borsboom (2006), the bias between observed and latent mean differences is also partly a pragmatic matter, depending on the purposes for which the scale is used. The CIP scale was developed for monitoring the effectiveness of teacher training programs in evidence-based ways from the undergraduate level onward during various phases of the teaching career (see also Ross and Bruce, 2007; Lucas and Villegas, 2013). The scale reflects confidence in culturally inclusive pedagogy with diverse children in general rather than any specific group such as special needs or immigrant children. The scale can be further expanded into a multidimensional scale by including more items on typical pedagogical practices, collaboration with team members and parents, and challenges of inclusive education (Lieber et al., 2008; Lai et al., 2016; Park et al., 2016; Gunn et al., 2021). The ECE teachers in Finnish daycare centers are required to provide each child with an individualized educational plan that is regularly revised to support the child’s optimal learning in mainstream early childhood classrooms. In line with the educational plans, the expanded CIP could include items on teacher’s confidence in supporting diverse children’s language and literacy, social–emotional competence, science, mathematics, and technology skills by employing play, reading, music, drama, craft, and outdoor activities. From the perspective of life-long learning, embedding more relational training and practice into curriculum design can have beneficial influences on pre- and in-service teachers’ confidence in implementing inclusive practice and developing high-quality teacher–child relationships in mainstream ECE classrooms.

## Data Availability Statement

The raw data supporting the conclusions of this article will be made available by the authors, without undue reservation.

## Ethics Statement

The studies involving human participants were reviewed and approved by the Ethics Committee for Human Sciences at the University of Turku. The patients/participants provided their written informed consent to participate in this study.

## Author Contributions

WY and MS contributed to the study design, the data collection and analysis, discussion of the results, and took major responsibility for writing the manuscript. EL provided advices on the statistical analyses. All authors contributed to the final version of the manuscript and approved the submitted version.

## Funding

This work was supported by the Ministry of Education and Culture of Finland under grant number OKM/66/523/2017.

## Conflict of Interest

The authors declare that the research was conducted in the absence of any commercial or financial relationships that could be construed as a potential conflict of interest.

## Publisher’s Note

All claims expressed in this article are solely those of the authors and do not necessarily represent those of their affiliated organizations, or those of the publisher, the editors and the reviewers. Any product that may be evaluated in this article, or claim that may be made by its manufacturer, is not guaranteed or endorsed by the publisher.

## Footnotes

^1^ https://www.european-agency.org/projects/iece

^2^Mplus constrains by default the factor loading of the first, so-called marker indicator to 1 to define the scale of the factor. The invariance testing supported the equality of the marker indicator loadings across the student teachers and qualified teachers of the CIP scale [*χ*^2^(1) = 2.31; *p* = 0.128] and of the STRS-SF scale, Closeness and Conflict [*χ*^2^(2) = 5.36, *p* = 0.069].
